# Serum-Based Diagnosis of Pediatric Tuberculosis by Assay of Mycobacterium tuberculosis Factors: a Retrospective Cohort Study

**DOI:** 10.1128/JCM.01756-20

**Published:** 2021-01-21

**Authors:** Yifan He, Christopher J. Lyon, Duc T. Nguyen, Chang Liu, Wei Sha, Edward A. Graviss, Tony Y. Hu

**Affiliations:** a Department of Biochemistry and Molecular Biology Center for Cellular and Molecular Diagnosis, School of Medicine, Tulane University, New Orleans, Louisiana, USA; b Shanghai Clinical Research Center for Infectious Diseases (Tuberculosis), Shanghai, China; c Department of Pathology and Genomic Medicine, Houston Methodist Research Institute, Houston, Texas, USA; d Department of Chemical Engineering, Biomedical Engineering Program, College of Engineering and Computing, University of South Carolina, Columbia, South Carolina, USA; St. Boniface Hospital

**Keywords:** extrapulmonary TB, Nanodisk-MS, pediatric TB, serum-based assay

## Abstract

Diagnosis of pediatric tuberculosis (TB) is often complicated by its nonspecific symptoms, paucibacillary nature, and the need for invasive specimen collection techniques. However, a recently reported assay that detects Mycobacterium tuberculosis virulence factors in serum can diagnose various TB manifestations, including paucibacillary TB cases, in adults with good sensitivity and specificity. The current study examined the ability of this M. tuberculosis biomarker assay to diagnose pediatric TB using archived cryopreserved serum samples drawn from children ≤18 years of age who were screened for suspected TB as part of a prospective population-based active surveillance study. In this analysis, any detectable level of either of the M. tuberculosis virulence factors CFP-10 and ESAT-6 was considered direct evidence of TB. Serum samples from 105 children evaluated for TB (55 TB cases and 50 close contacts without TB) were analyzed. The results of this analysis yielded sensitivity of 85.5% (95% confidence interval [CI], 73.3 to 93.5). Similar diagnostic sensitivities were observed for culture-positive (87.5%; 95% CI, 67.6 to 97.3) and culture-negative (83.9%; 95% CI, 66.3 to 94.5) TB cases and for culture negative pulmonary (77.8%; 95% CI, 40.0 to 97.2) and extrapulmonary (86.4%; 95% CI, 65.1 to 97.1) TB cases. These results suggest that serum biomarker analysis holds significant promise for rapid and sensitive diagnosis of pediatric TB cases, including extrapulmonary or paucibacillary TB cases. The ability to use frozen samples for this analysis should also permit assays to be performed at central sites, without a requirement for strict timelines for sample analysis.

## Abstract (precis)

Diagnosis of pediatric tuberculosis (TB) is often complicated by its nonspecific symptoms, paucibacillary nature, and the need for invasive specimen collection techniques. However, a recently reported assay that detects Mycobacterium tuberculosis virulence factors in serum can diagnose various TB manifestations, including paucibacillary TB cases, in adults with good sensitivity and specificity.

## INTRODUCTION

Detection and treatment of all tuberculosis (TB) cases are urgent operational priorities of TB control programs, particularly in high-burden countries. This is notoriously difficult in children, however, as many pediatric TB cases are asymptomatic or exhibit nonspecific symptoms and are frequently associated with paucibacillary or extrapulmonary infections (1). Children also have difficulty in producing sputum, and collection of sputum from infants and young children is generally not considered feasible without the use of invasive methods (2). Further, the proportion of TB cases that represent extrapulmonary disease and that thus require invasive tissue biopsies, often from more than one anatomical site, to obtain diagnostic samples is higher in children than in adults (3).

Due to the limitations of diagnostic approaches for pediatric TB, recommendations for new TB diagnostics emphasize the need for rapid biomarker-based assays that utilize nonsputum samples (4). Studies have focused either on detecting a Mycobacterium tuberculosis-specific immune response induced after M. tuberculosis infection or on directly detecting M. tuberculosis-derived factors present in the circulation. Efforts to develop serological tests for M. tuberculosis infection are ongoing, but conventional immunoassays have thus far failed to demonstrate the sensitivity and specificity required for clinical utility, potentially due to low circulating biomarker levels and/or interactions that serve to mask such biomarkers (5). To address these issues, our group recently developed an approach in which diagnostic serum samples are subjected to trypsin digestion to disrupt potential protein-protein interactions, immunoprecipitated to enrich specific biomarker peptides, and then analyzed by mass spectrometry (MS) to detect these biomarker targets (6). Serum CFP-10 and ESAT-6 (SCE) levels detected by this approach can serve as evidence of active TB and can accurately diagnose active TB cases in adults, independently of disease site or M. tuberculosis culture status (6–8), but the diagnostic performance of this method has not been rigorously evaluated in a pediatric population. The current study was therefore designed to evaluate the ability of this approach to diagnose active TB cases in children with suspected TB disease. Results of this study indicated that serum detection of two M. tuberculosis virulence factors had strong diagnostic performance for pediatric TB and exhibited similar levels of performance regardless of M. tuberculosis culture status or the M. tuberculosis infection site.

## MATERIALS AND METHODS

### Ethics statement.

The study protocol was approved by the biomedical research ethics committees of the City of Houston Department of Health and Human Services, Harris County Public Health and Environmental Services, and the Houston Methodist Research Institute.

### Study conduct and oversight.

Samples and clinical data analyzed in this study were obtained from archived study data and clinical specimens provided by investigators who conducted the Houston Tuberculosis Initiative (HTI) study (9), a retrospective population-based active surveillance and molecular epidemiology project that enrolled patients with suspected TB cases who were reported to the City of Houston Department of Health and Human Services and Harris County Public Health and Environmental Services from 20 October 1995 through 19 September 2002.

Serum samples and clinical data utilized in this cohort study were drawn from a pediatric subset of the HTI cohort. All children who were ≤18 years of age at HTI enrollment were eligible for inclusion in the current study; children were excluded on the basis of inconclusive diagnosis or insufficient serum volume (≥600 ml) for triplicate analysis or lipid contamination. Pediatric HTI study participants or their parents or legal guardians provided written informed consent for study participation before a child was enrolled in the study.

The HTI study was approved by the Institutional Review Board of Baylor College of Medicine, Houston, TX, and affiliated hospitals and by the University of Texas Health Science Center—Houston Committee for the Protection of Human Subjects.

### Classification of study subjects.

Children enrolled in the study were divided into three groups using established criteria (10). Children were identified as “confirmed TB” cases if they had a positive M. tuberculosis culture result. Children were identified as “unconfirmed TB” cases if they lacked a positive culture result but met two or more of the following criteria: (i) they exhibited a clinical course consistent with TB, (ii) they had close TB exposure or a positive tuberculin skin test (TST) result consistent with TB exposure (>5-mm area of induration), or (iii) they demonstrated clinical improvement upon treatment with ≥2 anti-TB drugs (10). Children who did not meet the criteria for confirmed TB or unconfirmed TB were designated to belong to a “non-TB” cohort, since they were not analyzed by M. tuberculosis culture and thus could not be classified as “unlikely TB” cases using the 2015 NIH diagnostic criteria. Children were considered to have pulmonary TB (PTB)-only disease manifestations if M. tuberculosis bacilli were isolated only from respiratory specimens (sputum, gastric aspirate, bronchus, bronchial fluid, or lung tissue) and/or if the lung was determined to be the sole site of disease based on all clinical evidence. Children were considered to have any extrapulmonary TB (EPTB) manifestation if there was evidence that the infection affected an extrapulmonary site, with or without affecting the respiratory tract, or if M. tuberculosis bacilli were isolated from nonrespiratory clinical specimens (e.g., pleural fluid, intrathoracic lymph nodes). Children with non-TB cases were close contacts of TB cases, were not analyzed by M. tuberculosis culture or acid-fast bacillus (AFB) smear, did not exhibit chest radiograph abnormalities or TB-specific signs/symptoms, and were ruled out as TB cases by a TB specialist after clinical evaluation. All children were tracked from their enrollment to the completion of the HTI study in 2004, a minimum of 2 years. No children assigned non-TB diagnoses demonstrated evidence of TB during this period.

### Serum CFP-10 and ESAT-6 (SCE) analysis.

Demographic information, medical history, results of radiological tests, and results of HIV tests (when part of routine care) were gathered from data recorded at HTI enrollment and during subsequent record review. All sample preparation and handling steps were conducted in a designated biosafety hood, following standard biosafety protocols for unfixed human blood samples.

Nanodisc particles were functionalized with peptide-specific CFP-10 and ESAT-6 antibodies and were then incubated with digested serum samples with constant rotary mixing to capture CFP-10 and ESAT-6 target peptides, after which peptide-loaded nanodisc particles were directly spotted on the target for matrix-assisted laser desorption ionization–time of flight mass spectrometry (MALDI-TOF MS) detection (6). CFP-10 and ESAT-6 levels were evaluated by analyzing these samples for the presence of monovalent CFP-10 and ESAT-6 peptide ions (*m*/*z* 1,593.75 and 1,900.95, respectively) and were quantified by measuring the intensity ratio of these peptide peaks against a constant amount of synthetic, isotope-labeled CFP-10 and ESAT-6 peptide (*m*/*z* 1,603.60 and 1,910.80) spiked into each sample. The individuals performing these assays were blind to the diagnosis and to other results associated with each sample. CFP-10 or ESAT-6 peptide signal above the limit of quantification was considered diagnostic for TB.

### Statistical analysis.

Demographic and clinical data were reported as frequencies and proportions for categorical variables and as median and interquartile range (IQR) for continuous variables. Differences between groups were determined by chi-square test or Fisher’s exact test for categorical variables and Kruskal-Wallis test for continuous variables as appropriate. Diagnostic sensitivity was analyzed for all tests, but specificity, positive predictive value (PPV), negative predictive value (NPV), and area under the concentration-time curve (AUC) were evaluated only for SCE assay and TST results, since the non-TB group lacked M. tuberculosis culture and smear results. All analyses were performed using Stata version 15.1 (StataCorp LLC, TX), and *P* values of <0.05 were considered statistically significant.

## RESULTS

### Study population demographics and clinical criteria.

**FIG 1:**
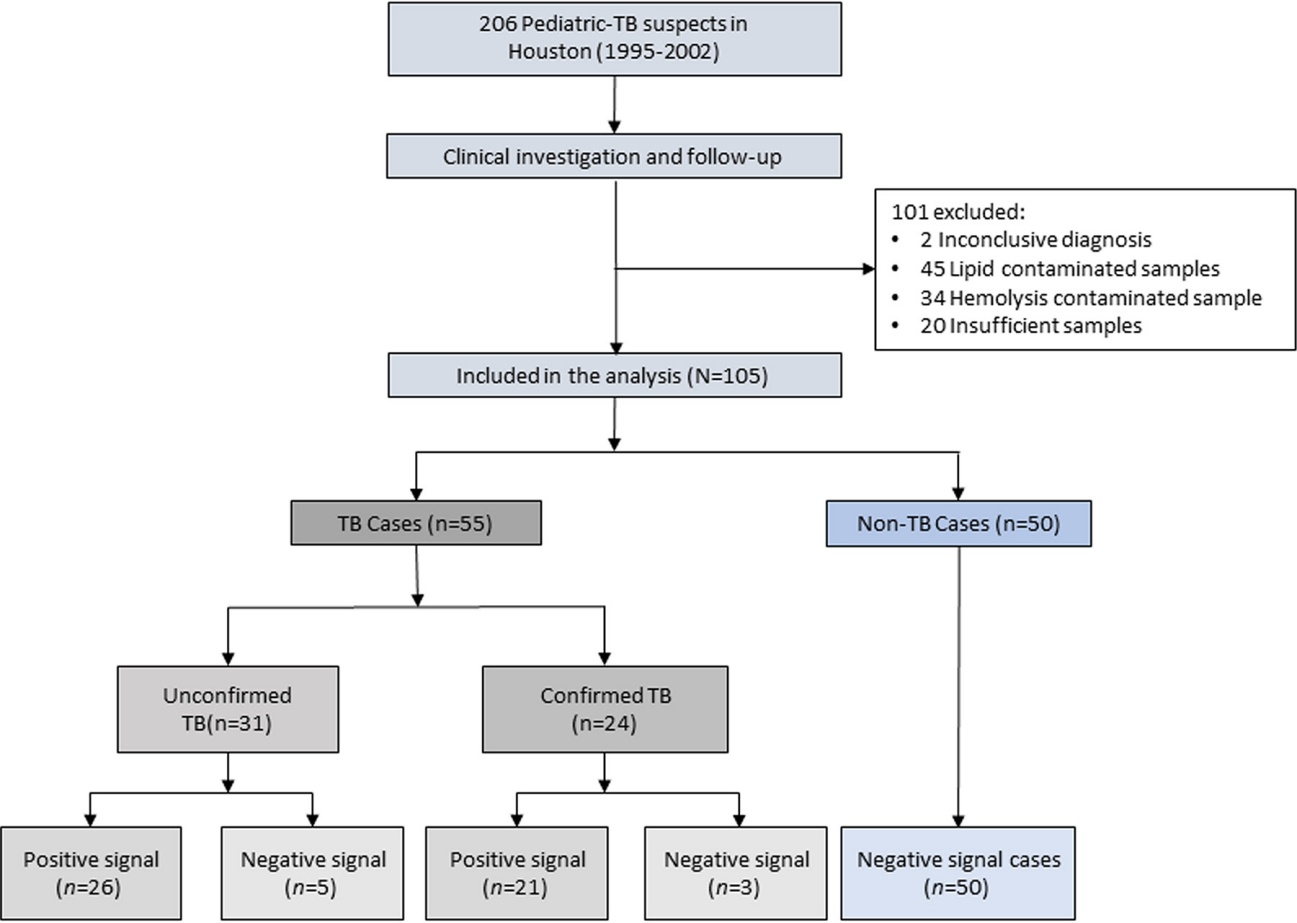
The study participants who were enrolled were children in the HTI cohort with available serum samples. Children were segregated into a confirmed TB case group and an unconfirmed TB case group based on an accepted clinical case definition (10). Children assigned to the non-TB cohort were close contacts of TB cases who did not exhibit chest radiograph abnormalities or TB-specific signs/symptoms and who were ruled out for TB by a TB specialist after clinical evaluation but were not evaluated by M. tuberculosis culture and thus could not be classified as unlikely TB cases using the clinical algorithm used to assign children to the confirmed TB and unconfirmed TB cohorts. Children were judged to be signal positive for the CFP-10 and/or ESAT-6 target peptides if either peptide was detected above its limit of detection or were otherwise judged to be signal negative.

Samples and clinical data analyzed in this study were drawn from 206 children with suspected TB cases who enrolled in the HTI cohort between 1995 and 2002, of which 105 met the criteria for serum biomarker analysis (Fig. 1). This group contained 24 confirmed TB cases, 31 unconfirmed TB cases, and 50 non-TB cases (see Table S1 in the supplemental material). Detected sites of extrapulmonary involvement among the 30 EPTB cases identified in our study were found in the lymph nodes (24 cases), pleura (2 cases), hip joint (2 cases), meninges (1 case), and peritoneum (1 case). None of the PTB-only cases demonstrated any evidence of EPTB symptoms. Enrolled children had a median age of 9.0 years and were primarily Hispanic (63.8%), similarly split by gender (51.4% male), and predominantly HIV negative (1 HIV-positive child). None of these factors differed between the TB and non-TB groups except ethnicity and age distribution. Children with TB had a history of previous TB and were more likely to have a positive TST result than children with non-TB diagnoses. A difference in Mycobacterium bovis BCG vaccination rates was rendered insignificant when these groups were stratified by foreign birth. BCG vaccination is recommended for children in high-TB-burden countries, but data were not available to determine if the TB group contained more children from high-TB-burden countries.

Most TB cases (56.4%) were clinically diagnosed, but no differences in age, gender, or ethnicity were detected among the confirmed TB and unconfirmed TB cases. Nor did these groups differ by most measures of TB risk, except in their history of close TB contact and the prevalence of any form of EPTB, which were both more common in the clinically diagnosed group.

### TB screening and diagnostic test results.

All children enrolled in the parent HTI study were expected to receive a TST as part of normal evaluation and the study protocol. TST records were obtained for most (93.3%) children (Table S2), and positive TST results were significantly more common in the TB group than in the non-TB group (76% versus 30%). M. tuberculosis culture demonstrated better diagnostic sensitivity than AFB smear (43.6% versus 12.7%) but failed to diagnose most TB cases. Serum CFP-10 and/or ESAT-6 levels were detected for most TB cases (85.5%) but serum CFP-10 or ESAT-6 or both were undetectable in all the non-TB cases, including 15 children with positive TST results who may have had latent TB infections (Table S2).

### Serum biomarker levels and diagnostic performance for different TB subtypes.

**FIG 2:**
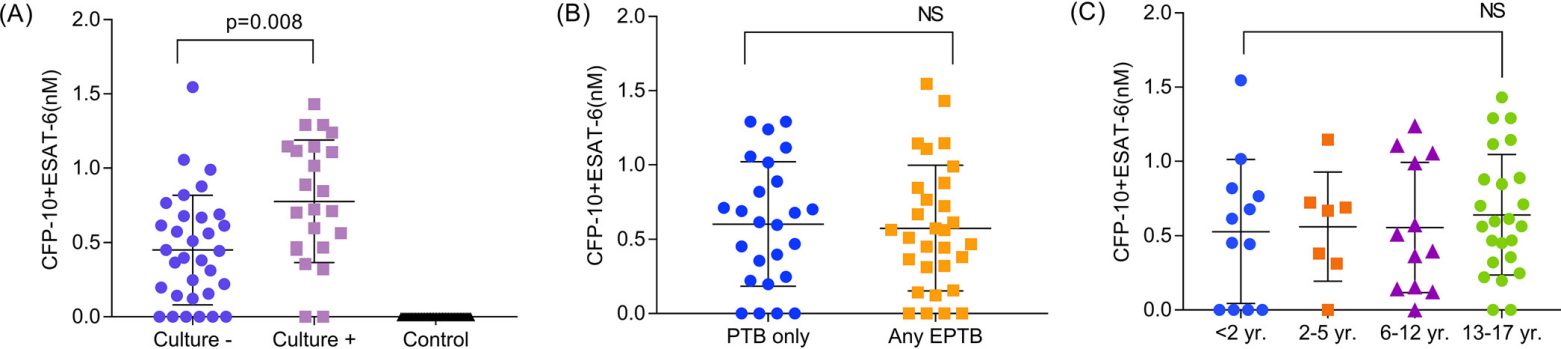
Combined serum CFP-10 and ESAT-6 concentration (CFP-10+ESAT-6) (A) in culture-positive (confirmed) and culture-negative (unconfirmed) TB cases and control (non-TB) subjects; (B) in PTB-only and any EPTB cases; and (C) in all TB cases, stratified by age. Differences between different groups were determined using the Kruskal Wallis test. NS = no significant difference.

Serum CFP-10 and/or ESAT-6 signals were observed in most confirmed TB (87.5%) and unconfirmed TB (83.9%) cases (Table 1), indicating that children with paucibacillary M. tuberculosis culture specimens were not diagnosed with reduced efficiency despite the significant differences in CFP-10 and ESAT-6 levels between these groups (Fig. 2A). The small sample sizes prevented conclusive analysis of whether the SCE diagnostic sensitivities differed for confirmed TB cases with AFB-positive results (100%; 95% CI, 59.0 to 100) versus AFB-negative results (82.4%; 95% CI, 56.6 to 96.2) or for children with PTB-only cases (84%; 95% CI, 63.9 to 95.5) versus any EPTB cases (86.4%; 95% CI, 65.1 to 97.1) (Table 1). Serum biomarker levels, however, did not differ between PTB and EPTB cases (Fig. 2B). M. tuberculosis cultures exhibited 64.0% (95% CI, 42.5 to 82.0) sensitivity for PTB-only cases and 26.7% (95% CI, 12.2 to 45.9) sensitivity for cases with any EPTB disease, while AFB smear results revealed 24.0% (95% CI, 9.4 to 45.1) and 3.3% (95% CI, 8.0 to 17.2) sensitivity for these cases, respectively.

**TABLE 1:** Sensitivity and specificity of the SCE assay for indicated TB groups and subgroups^*a*^

Group (no. of AFB-positive cases/ total no. of cases)	% sensitivity (95% CI)		
	Total TB cases (*N* = 55)	AFB-positive cases (*n* = 7)	AFB-negative cases^*b*^ (*n *=* *44)
All TB cases (7/55)	85.5 (73.3–93.5)	100 (59.0–100)	84.1 (69.9–93.4)

Confirmed TB (7/24)	87.5 (67.6–97.3)	100 (59.0–100)	82.4 (56.6–96.2)
PTB only (6/16)	87.5 (61.7–98.4)	100 (54.1–100)	80.0 (44.4–97.5)
Any EPTB (1/8)	87.5 (47.3–99.7)	100 (2.5–100)	85.7 (42.1–99.7)

Unconfirmed TB (0/31)	83.9 (66.3–94.5)	NA	85.2 (66.3–95.8)
PTB only (0/9)	77.8 (40.0–97.2)	NA	75.0 (34.9–99.7)
Any EPTB (0/22)	86.4 (65.1–97.1)	NA	87.9 (65.3–98.6)

Non-TB subjects (*N* = 50)	100 (92.9–100)	NA	NA

a 95% CI, 95% confidence interval; NA, not available.

b No AFB results were available for 4 TB subjects.

### Serum biomarker levels and diagnostic performance in children of different ages.

Changes that occur during normal pediatric development, particularly those affecting immune responses, may also influence the performance of TB diagnostics and have the potential to alter the levels of pathogen-derived factors present in the circulation. Serum biomarker levels did not appear to differ with age (Fig. 2C), implying that circulating biomarker levels or biomarker detection was not inhibited by such potential changes. SCE results revealed better overall diagnostic sensitivity than any other diagnostic available for comparison in this cohort, although overall performance could not be addressed due to the lack of culture and smear data for the non-TB group, and age-specific differences were unclear due to limited sample size (Table 2; see also Table S3).

**TABLE 2:** Sensitivity of the methods stratified by age^*a*^

Method	TB cases (**+**/N)	Non-TB subjects (**+**/*N*)	% sensitivity (95% CI)
SCE	47/55	0/50	85.5 (73.3–93.5)
TST	42/48	15/50	87.5 (74.8–95.3)
M. tuberculosis culture	24/55	—^*b*^/50	43.6 (30.3–57.7)
AFB smear	7/55	—/50	12.7 (5.3–24.5)

a +/*N*, number of subjects with positive results/total number of subjects; 95% CI, 95% confidence interval; SCE, serum CFP10/ESAT-6.

b —, no test results available (given that M. tuberculosis culture and AFB smear were not done for children judged not to have TB).

### Diagnostic performance of different diagnostic models.

Different combinations of diagnostic assay data were also analyzed to determine if composite results offered any potential for improved diagnostic performance (Table 3). A model that employed M. tuberculosis culture and SCE results exhibited the best overall performance; however, given the time required to obtain final M. tuberculosis culture results, the additional cases that might be diagnosed by this dual approach would be recognized much later than those diagnosed by the serum data. A composite model that used TST and SCE data, which would permit more-rapid diagnosis, demonstrated superior diagnostic sensitivity.

**TABLE 3:** Diagnostic accuracies of all methods compared to the composite reference standard^*a*^

Method	TB cases (**+**/*N*)	Non-TB subjects (**+**/*N*)	% sensitivity (%, 95% CI)
One parameter			
Smear	6/48	—^*b*^/50	12.5 (4.7–25.2)
Culture	19/48	—/50	39.6 (25.8–54.7)
TST	42/48	15/50	87.5 (74.8–95.3)
SCE	40/48	0/50	83.3 (69.8–92.5)

Two parameters			
Smear + culture	19/48	—/50	39.6 (25.8–54.7)
Smear + TST	42/48	15/50	87.5 (74.8–95.3)
Smear + SCE	40/48	0/50	83.3 (69.8–92.5)
Culture + TST	43/48	15/50	89.6 (77.3–96.5)
Culture + SCE	43/48	0/50	83.3 (69.8–92.5)
TST + SCE	46/48	15/50	95.8 (85.8–99.5)

Three parameters			
Smear + culture + TST	43/48	15/50	89.6 (77.3–96.5)
Smear + culture + SCE	43/48	0/50	83.3 (69.8–92.5)
Culture + TST + SCE	46/48	15/50	83.3 (69.8–92.5)
Smear + TST + SCE	46/48	15/50	95.8 (85.8–99.5)

a *N*, total number of subjects; 95% CI, 95% confidence interval; SCE, serum CFP10/ESAT-6.

b —, no test results available (given that M. tuberculosis culture and AFB smear were not done for children judged not to have TB).

## DISCUSSION

Detection of M. tuberculosis-derived virulence factors in serum provides direct evidence of active TB, but these factors may circulate at low concentrations or form nonspecific interactions or antibody-antigen complexes to limit detection by standard immunoassay. The SCE assay employed in this study utilizes trypsin to digest diagnostic serum samples to disrupt interactions that could mask biomarker detection. Sensitivity for low-abundance biomarkers is addressed by using immunoprecipitation to concentrate peptide biomarkers prior to MS analysis. Specificity is conferred by the peptide capture antibodies and the characteristic mass/charge ratio of each biomarker peptide, and the sequence of detected peptides can be confirmed by tandem MS analysis.

Serum CFP-10 and ESAT-6 levels were analyzed as biomarkers of pediatric TB since serum levels of these proteins are diagnostic for adult PTB and EPTB (6), because both proteins are secreted by M. tuberculosis to promote immunopathologic responses, and because the loss of either protein reduces M. tuberculosis virulence (11, 12). Previous studies have employed enzyme-linked immunosorbent assays (ELISAs) to detect CFP-10 and ESAT-6 (13, 14) or MPT-64 (15–17) for TB diagnosis, primarily in nonserum samples, but most of those reports lack follow-up studies to validate their results or to evaluate the utility of these assays for EPTB diagnosis.

SCE assay analysis revealed 85.5% overall sensitivity for confirmed TB and unconfirmed TB cases and 100% specificity to exclude individuals with non-TB diagnoses. SCE sensitivity was superior to M. tuberculosis culture (43.6%) and AFB smear (12.7%) sensitivity, and the latter results were similar to results reported in other pediatric studies (18–22). The time required for SCE assay performance (∼4 h) was shorter than that required for M. tuberculosis culture (3 to 8 weeks) and for rapid liquid M. tuberculosis culture systems, which require on average 7 to 9 days to obtain results from AFB smear-positive samples (23). SCE assay performance times were comparable to those seen with AFB smear assays, but the SCE assays had better sensitivity, while TST provided diagnostic sensitivity comparable to SCE results but had lower specificity (70.0% versus 100%) and longer turn around (days versus hours).

The WHO recommends Xpert MTB/RIF as the initial diagnostic for adults and children with suspected PTB and EPTB cases but has acknowledged the reduced quality of evidence for its use in EPTB diagnosis (24). Pediatric TB is frequently paucibacillary and/or extrapulmonary, resulting in reduced sensitivity of detection by current diagnostics (25, 26). The samples analyzed in this study predated Xpert adoption, and archived sputum and tissue biopsy samples were not available, precluding direct comparison of Xpert and SCE diagnostic performances. However, results from one recent meta-analysis indicate that Xpert has reduced sensitivity with sputum samples (62%) and gastric lavage samples (66%) from culture-positive pediatric PTB cases relative to sputum samples (98%) from culture-positive adult PTB cases (27). Culture-positive pediatric PTB is detected with improved sensitivity by Xpert Ultra versus Xpert (64% versus 54%) (28), but there are no data on their relative levels of performance for pediatric EPTB diagnosis.

Xpert sensitivity for adult EPTB diagnosed by M. tuberculosis culture is reduced when using cerebrospinal fluid samples (CSF) (80·5%) and pleural fluid samples (46.4%) (29) and is further reduced when using CSF samples (62·8%) and pleural fluid samples (21.4%) from paucibacillary adult EPTB cases diagnosed by a composite reference standard (29). SCE data, however, showed similar levels of sensitivity for confirmed TB cases (87.5%) and unconfirmed TB cases (83.9%) in children and for PTB and EPTB cases within these groups, and the data were comparable to results reported for adults (6, 8). SCE assay may thus have superior diagnostic performance for pediatric PTB and EPTB, independently of M. tuberculosis culture status. This study was not powered to address diagnostic differences, if any, for EPTB at different anatomical sites. However, SCE results diagnosed one patient with TB meningitis, which is difficult to diagnose by conventional methods and requires a rapid intervention to prevent high mortality.

EPTB is more common in children, partly due to the reduced ability of their developing immune systems to contain M. tuberculosis bacilli in pulmonary granulomas (30). Current WHO recommendations for EPTB diagnosis suggest that several invasive procedures should be performed to obtain diagnostic specimens, including a lumber puncture, a pleural tap, and a lymph node biopsy or use of a fine needle to obtain aspirate. Use of a small peripheral blood sample would greatly simplify pediatric EPTB diagnosis.

It is generally accepted that young children are the most difficult to diagnose, due to the difficulty of obtaining diagnostic samples, which may have very low M. tuberculosis concentrations (1). SCE diagnostic sensitivity exhibited a potential decrease in the members of the youngest TB subgroup, all of whom were less than 1 year of age. Young children demonstrate decreased or aberrant immune responses compared to adults or teenagers, which may permit the development of paucibacillary M. tuberculosis infections with correspondingly low serum CFP-10 and ESAT-6 levels. However, the levels of SCE assay performance were similar between culture-positive and culture-negative and PTB and EPTB cases in this study, suggesting that neither bacterial load nor infection site may explain this potential difference.

Composite models generated using data from multiple assays found that AFB smear data did not improve TST or SCE diagnostic performance. It is not clear if TST data can increase SCE diagnostic sensitivity at the cost of specificity due to the confidence intervals of these assays, and addition of culture and/or smear data to TST and SCE data had no apparent benefit.

The SCE assay employed in this study has several advantages, as it does not require bacterial isolation; requires only a small peripheral blood sample that can be safely drawn from all individuals, including infants; has high sensitivity and specificity for culture-negative pediatric PTB and EPTB cases; uses a streamlined process amenable to high-throughput operation in clinical settings; can be performed using equipment already approved by the Food and Drug Administration for other clinical diagnostic assays; and can use frozen serum samples, allowing samples to be transported to and analyzed at central testing sites without any restriction on the sample-to-testing time frame required to obtain valid assay results.

This study had several limitations. First, it employed cryopreserved serum obtained from a study not designed to evaluate TB diagnostics, although demographic, clinical, and TB classification results determined in this study allowed a *post hoc* serum analysis. Serum CFP-10 and ESAT-6 levels are stable during extended storage at −80°C (8); however, sera analyzed in this study were archived at −80°C for >17 years prior to analysis and thus could have experienced oxidation or degradation during this extended interval to reduce detection efficiency and diagnostic sensitivity versus what might be observed with fresh samples. Second, this study was unable to compare SCE results to results from molecular methods (e.g., Xpert) that were not available during the initial study, although Xpert MTB/RIF and Xpert MTB/RIF Ultra are not superior to M. tuberculosis culture in children (28). Third, the small sample size limited the power to distinguish potential diagnostic sensitivity differences between PTB and EPTB cases and by age group, particularly in children ≤5 years of age. No differences could be evaluated by HIV status, since only one child with TB was HIV positive. Large-scale studies are required to address potential diagnostic differences among these groups. Finally, the lack of microbiological data in the non-TB group analyzed in this study prevented a full comparison of the diagnostic performance of our SCE assay to that of M. tuberculosis culture, the gold standard for TB diagnosis.

The SCE assay is compatible with MALDI-TOF MS systems used by hospitals and public health laboratories for microbial identification, but MS represents a significant barrier for TB diagnosis in resource-limited settings. However, SCE analysis can be adapted to less-expensive, portable MS platforms or to other sensitive and cost-effective assay systems. Serum samples analyzed in this study were excluded for excessive hemolysis or lipid contamination, since each can reduce the sensitivity of MALDI-TOF MS analyses. Refining the serum processing procedure to remove this interference would facilitate clinical adoption.

Direct measurement of M. tuberculosis-secreted CFP-10 and ESAT-6 expression in patient serum samples successfully diagnosed all forms of pediatric TB with high diagnostic sensitivity and specificity in this study. Notably, this method can diagnose patients who cannot produce useful respiratory samples or who have suspected EPTB cases that would otherwise require tissue biopsies or other invasive procedures to obtain diagnostic specimens. While further studies are required to confirm these findings in additional pediatric populations, this approach appears to hold great promise for rapid diagnosis of pediatric TB in well-equipped clinical laboratories. Broad adoption of this assay approach, however, will require translation of the current assay to less-expensive analysis platforms suitable for use in resource-limited settings.

## Supplementary Material

Supplemental file 1
