# Supplementary material for: Serum-Based Diagnosis of Pediatric Tuberculosis by Assay of Mycobacterium tuberculosis Factors: a Retrospective Cohort Study
Source: J Clin Microbiol. 2021 Jan 21;59(2):e01756-20. doi: 10.1128/JCM.01756-20 (PMC8111146; doi:10.1128/JCM.01756-20)
Supplement: Supplemental file 1 [file JCM.01756-20-s0001.pdf]

Supplementary Material

Group Characteristic	All participants (N=105)				TB cases only (n=55)		
	**Total (N=105)**	**Non-TB subjects (n=50)**	**TB cases (n=55)**	**P value**	**Confirmed TB (n=24)**	**Unconfirmed TB (n=31)**	**P value**
Age in years, median (IQR)	9·0 (5·0, 14·0)	9·0 (5·0, 12·0)	10·0 (2·0, 15·0)	0·56	15·0 (2·5, 16·0)	9·0 (2·0, 14·0)	0·14
<2 yr.	15 (14·3)	3 (6·0)	12 (21·8)	0·02	5 (20·8)	7 (22·6)	0·88
2-5 yr.	15 (14·3)	10 (20·0)	5 (9·1)	0·11	2 (8·3)	3 (9·7)	0·86
6-12 yr.	38 (36·2)	25 (50·0)	13 (23·6)	0·01	3 (12·5)	10 (32·3)	0·09
13-17 yr.	37 (35·2)	12 (24·0)	25 (45·5)	0·02	14 (58·33)	11 (35·5)	0·09
Male gender	54 (51·4)	25 (50·0)	29 (52·7)	0·85	11 (45·8)	18 (58·1)	0·42
Ethnicity, Hispanic	67 (63·8)	27 (54·0)	40 (72·7)	0·046	20 (83·3)	20 (64·5)	0·12
HIV (+) status	1 (1·0)	0	1 (1·8)	1·00	1 (4·2)	0	0·44
BCG vaccination	12 (11·4)	2 (4·0)	10 (18·2)	0·03	6 (25·0)	4 (12·9)	0·30
History of close TB contact	66(65.3)	50(100)	16 (29·1)	<0·001	2 (8·3)	14 (45·2)	0·003
History of previous TB	52 (49·5)	0	52 (94·5)	<0·001	22 (91·7)	30 (96·8)	0·57
Positive TST	57 (54·3)	15 (30·0)	42 (76·4)	<0·001	18 (75·0)	24 (77·4)	1·00
Abnormal chest X-ray	28 (26·7)	0	28 (50·9)	<0·001	9 (37·5)	19 (61·3)	0·11
TB manifestation							0·007
PTB only	--	--	25 (45·5)	--	16 (66·7)	9 (29·0)	
Any EPTB	--	--	30 (54·5)	--	8 (33·3)	22 (71·0)	

Supplementary Table 1 **:** *Demographics and clinical characteristics of the paediatric study population.*
Note: Values indicate number positive (%) unless otherwise indicated; IQR, interquartile range; BCG, Bacille Calmette-Guerin; TST, tuberculin skin test; PTB, pulmonary TB; EPTB= Extrapulmonary TB; --, not applicable.

	Total (N=105) n (%)	Non-TB (N=50) n (%)	TB (N=55) n (%)	P-value
TST				<0·001
Negative	41 (39·0)	35 (70·0)	6 (10·9)	
Positive	57 (54·3)	15 (30·0)	42 (76·4)	
Unknown/Not done	7 (6·7)	0	7 (12·7)	
AFB smear				<0·001
Negative	44 (41·9)	0	44 (80·0)	
Positive	7 (6·7)	0	7 (12·7)	
Not done	54 (51·4)	50 (100)	4 (7·3)	
Mtb culture				<0·001
Negative	31 (29·5)	0	31 (56·4)	
Positive	24 (22·9)	0	24 (43·6)	
Not done	50 (47·6)	50 (100)	0	
SCE				<0·001
Negative	58 (55·2)	50 (100)	8 (14·5)	
Positive	47 (44·8)	0	47 (85·5)	

Supplementary Table 2: *TB tests with "positive", "negative" and "unknown/not done" results.*
N, total number of subjects; n, number of test results.

Method	TB cases (+/N)	Non-TB subjects (+/N)	Sensitivity % (95% CI)	Specificity % (95% CI)	PPV % (95% CI)	NPV% (95% CI)	ROC AUC
**SCE**	**47/55**	**0/50**	**85·5 (73·3, 93·5)**	**100 (92·9, 100)**	**100 (92·5, 100)**	**86·2 (74·6, 93·9)**	**0·93**
<2 yr.	9/12	0/3	75 (42·8, 94·5)	100 (42·8, 100)	100 (66·4, 100)	50·0 (11·8, 88·2)	0·88
2-5 yr.	5/5	0/10	100 (47·8, 100)	100 (69·2, 100)	100 (47·8, 100)	100 (69·2, 100)	1·00
6-12 yr.	11/13	0/25	84·6 (54·6, 98·1)	100 (86·3, 100)	100 (71·5, 100)	92·6 (75·7, 99·1)	0·92
13-17 yr.	22/25	0/12	87·5 (67·6, 97·3)	100 (73·5, 100)	100 (83·9, 100)	80·0 (51·9, 95·7)	0·94
**TST**	**42/48**	**15/50**	**87·5 (74·8, 95·3)**	**70·0 (55·4, 82·1)**	**73·7 (60·3, 84·5)**	**85·4(70·8, 94·4)**	**0·73**
<2 yr.	10/11	0/3	90·9 (58·7, 99·8)	100 (29·2, 100)	100 (69·2, 100)	75·0 (19·4, 99·4)	0·95
2-5 yr.	3/3	3/10	100 (29·2, 100)	70·0 (34·8, 93·3)	50·0 (11·8, 88·2)	100 (59·0, 100)	0·85
6-12 yr.	11/13	9/25	84·6 (54·6, 98·1)	64·0 (42·5, 82·0)	55·0 (31·5, 76·9)	88·9 (65·3, 98·6)	0·74
13-17 yr.	18/21	3/12	85·7 (63·7, 97·0)	75·0 (42·8, 94·5)	85·7 (63·7, 97·0)	75·0 (42·8, 94·5)	0·80
***Mtb* Culture**	**24/55**	**--/50**	**43·6 (30·3, 57·7)**	/	/	/	/
<2 yr.	5/12	--/3	41·7 (15·2, 72·3)	/	/	/	/
2-5 yr.	2/5	--/10	40·0 (5·3, 85·3)	/	/	/	/
6-12 yr.	3/13	--/25	23·1 (5·0, 53·8)	/	/	/	/
13-17 yr.	14/25	--/14	56·0 (34·9, 75·6)	/	/	/	/
**AFB Smear**	**7/55**	**--/50**	**12·7 (5·3, 24·5)**	/	/	/	/
<2 yr.	1/12	--/3	8·3 (0·2, 38·5)	/	/	/	/
2-5 yr.	0/5	--/10	0 (0, 52·2)	/	/	/	/
6-12 yr.	2/13	--/25	15·4 (1·8, 45·4)	/	/	/	/
13-17 yr.	4/25	--/14	16·0 (4·5, 36·1)	/	/	/	/

***Supplementary Table 3*：** *Sensitivity of the methods stratified by age.*

Note: N, total number of subjects; 95% CI, 95% confidence interval; PPV, positive predictive values; NPV, negative predictive values; AUC, area under the Receiver operating characteristic (ROC) curve; SCE, Serum CFP10/ESAT-6; --, no test results available. Given that Mtb culture and AFB smear were not done for children judged not to have TB, Mtb culture and AFB smear were assumed to have zero false positive rates.
